# Supplementary material for: Multi‐dataset identification of innovative feature genes and molecular mechanisms in keratoconus
Source: J Cell Mol Med. 2024 Sep 19;28(18):e70079. doi: 10.1111/jcmm.70079 (PMC11412914; doi:10.1111/jcmm.70079)
Supplement: Supplementary file 2 — Material S2. [file JCMM-28-e70079-s002.zip › 7.GSEA analysis/1.ARL11/KEGG_COLORECTAL_CANCER.html]

Details for gene set KEGG\_COLORECTAL\_CANCER[GSEA]

|  || Dataset | ARL11.group.cls#h\_versus\_l |
| Phenotype | group.cls#h\_versus\_l |
| Upregulated in class | h |
| GeneSet | KEGG\_COLORECTAL\_CANCER |
| Enrichment Score (ES) | 0.21352775 |
| Normalized Enrichment Score (NES) | 0.8630456 |
| Nominal p-value | 0.6343874 |
| FDR q-value | 1.0 |
| FWER p-Value | 1.0 |
Table: GSEA Results Summary

  

Fig 1: Enrichment plot: KEGG\_COLORECTAL\_CANCER      
 Profile of the Running ES Score & Positions of GeneSet Members on the Rank Ordered List

  

| SYMBOL | TITLE | RANK IN GENE LIST | RANK METRIC SCORE | RUNNING ES | CORE ENRICHMENT || 1 | APC2 | na | 1889 | 0.305 | 0.0073 | Yes |
| 2 | PIK3R3 | na | 2180 | 0.284 | 0.0564 | Yes |
| 3 | TGFBR2 | na | 2250 | 0.279 | 0.1110 | Yes |
| 4 | PIK3CG | na | 2870 | 0.247 | 0.1432 | Yes |
| 5 | RAC3 | na | 4612 | 0.179 | 0.1292 | Yes |
| 6 | DCC | na | 5487 | 0.152 | 0.1347 | Yes |
| 7 | PIK3R1 | na | 6079 | 0.135 | 0.1451 | Yes |
| 8 | TGFB2 | na | 7601 | 0.100 | 0.1215 | Yes |
| 9 | PIK3R2 | na | 8783 | 0.078 | 0.1033 | Yes |
| 10 | FOS | na | 8788 | 0.078 | 0.1190 | Yes |
| 11 | SMAD2 | na | 8821 | 0.078 | 0.1338 | Yes |
| 12 | AKT2 | na | 9348 | 0.070 | 0.1327 | Yes |
| 13 | TP53 | na | 9447 | 0.068 | 0.1437 | Yes |
| 14 | CASP3 | na | 9607 | 0.066 | 0.1524 | Yes |
| 15 | PIK3R5 | na | 10118 | 0.059 | 0.1497 | Yes |
| 16 | RALGDS | na | 10287 | 0.056 | 0.1563 | Yes |
| 17 | MYC | na | 10444 | 0.054 | 0.1628 | Yes |
| 18 | SMAD4 | na | 10504 | 0.054 | 0.1720 | Yes |
| 19 | APPL1 | na | 10611 | 0.052 | 0.1795 | Yes |
| 20 | BRAF | na | 10628 | 0.052 | 0.1897 | Yes |
| 21 | KRAS | na | 10630 | 0.052 | 0.2002 | Yes |
| 22 | TCF7L2 | na | 10815 | 0.050 | 0.2050 | Yes |
| 23 | MSH3 | na | 11101 | 0.046 | 0.2062 | Yes |
| 24 | MAPK9 | na | 11167 | 0.046 | 0.2135 | Yes |
| 25 | JUN | na | 13319 | 0.024 | 0.1564 | No |
| 26 | PIK3CA | na | 13512 | 0.023 | 0.1555 | No |
| 27 | SMAD3 | na | 14170 | 0.017 | 0.1401 | No |
| 28 | CTNNB1 | na | 14653 | 0.014 | 0.1290 | No |
| 29 | MAPK10 | na | 15299 | 0.009 | 0.1122 | No |
| 30 | BIRC5 | na | 16081 | 0.003 | 0.0903 | No |
| 31 | RAC1 | na | 16390 | 0.001 | 0.0817 | No |
| 32 | MSH2 | na | 16407 | 0.001 | 0.0814 | No |
| 33 | CCND1 | na | 16546 | -0.000 | 0.0774 | No |
| 34 | BAX | na | 17601 | -0.008 | 0.0486 | No |
| 35 | CYCS | na | 17719 | -0.008 | 0.0469 | No |
| 36 | AKT1 | na | 18085 | -0.011 | 0.0386 | No |
| 37 | TCF7 | na | 18185 | -0.012 | 0.0380 | No |
| 38 | RAC2 | na | 18868 | -0.016 | 0.0216 | No |
| 39 | AXIN2 | na | 19144 | -0.018 | 0.0173 | No |
| 40 | BAD | na | 19342 | -0.019 | 0.0156 | No |
| 41 | TGFBR1 | na | 19919 | -0.023 | 0.0037 | No |
| 42 | CASP9 | na | 20358 | -0.026 | -0.0036 | No |
| 43 | GSK3B | na | 20728 | -0.029 | -0.0083 | No |
| 44 | MSH6 | na | 21102 | -0.032 | -0.0126 | No |
| 45 | RHOA | na | 21886 | -0.038 | -0.0274 | No |
| 46 | MAPK3 | na | 22614 | -0.046 | -0.0389 | No |
| 47 | MLH1 | na | 22834 | -0.048 | -0.0355 | No |
| 48 | RAF1 | na | 23336 | -0.054 | -0.0390 | No |
| 49 | PIK3CB | na | 23818 | -0.060 | -0.0407 | No |
| 50 | BCL2 | na | 24069 | -0.063 | -0.0351 | No |
| 51 | MAPK8 | na | 24525 | -0.070 | -0.0341 | No |
| 52 | TCF7L1 | na | 24530 | -0.070 | -0.0201 | No |
| 53 | MAPK1 | na | 24626 | -0.071 | -0.0084 | No |
| 54 | AXIN1 | na | 24756 | -0.073 | 0.0027 | No |
| 55 | TGFB1 | na | 25459 | -0.082 | -0.0009 | No |
| 56 | PIK3CD | na | 25759 | -0.087 | 0.0080 | No |
| 57 | ARAF | na | 26195 | -0.093 | 0.0144 | No |
| 58 | AKT3 | na | 27547 | -0.120 | -0.0003 | No |
| 59 | APC | na | 29306 | -0.166 | -0.0174 | No |
| 60 | MAP2K1 | na | 31070 | -0.223 | -0.0231 | No |
| 61 | LEF1 | na | 32575 | -0.292 | -0.0072 | No |
| 62 | TGFB3 | na | 33262 | -0.342 | 0.0423 | No |
Table: GSEA details [plain text format]

  

Fig 2: KEGG\_COLORECTAL\_CANCER      
 Blue-Pink O' Gram in the Space of the Analyzed GeneSet

  

Fig 3: KEGG\_COLORECTAL\_CANCER: Random ES distribution      
 Gene set null distribution of ES for **KEGG\_COLORECTAL\_CANCER**
